# Touch as emotion regulation

**DOI:** 10.3389/fpsyg.2025.1418374

**Published:** 2026-01-27

**Authors:** Özge Ugurlu, Dacher Keltner

**Affiliations:** Department of Psychology, University of California, Berkeley, Berkeley, CA, United States

**Keywords:** touch, affect, emotion regulation, emotional communication, stress buffer

## Abstract

Interpersonal touch is central to emotional well-being. Even though numerous studies demonstrate its efficacy in mitigating negative affect, emotional distress, and physical pain across human development and in diverse species, little has been offered regarding how touch functions as a form of emotion regulation. To address that gap, we first review how touch—enabled by an interpersonal tactile system—facilitates emotion regulation through its effects on neurophysiological responses. We then outline how touch communicates emotional states in interpersonal relationships that are vital to emotion regulation. Finally, we chart four pathways through which touch supports emotion regulation.

## Introduction

From the first moments of life, interpersonal touch can convey messages that are, at times, more powerful than language (Frank, 1957; Jones and Yarbrough, 1985; McDaniel and Andersen, 1998). Such tactile contact is vital for the formation and maintenance of social relationships (Jablonski, 2021), the communication of distinct emotions (Hertenstein et al., 2006), and the regulation of stress (Fotopoulou et al., 2022).

Below, we detail how touch functions as a form of emotion regulation, defined as goal-directed processes that influence the intensity, duration, and type of emotions experienced (Gross and Thompson, 2007). Despite the central role of touch in emotion communication and stress regulation, this line of thinking has not been developed before. To make this argument, we first outline the neurophysiology of interpersonal touch and the ways in which we ascribe meaning to it. We then consider the broader functions of touch documented in empirical studies. Finally, we conclude with four ways in which touch supports emotion regulation.

## The neurophysiology of interpersonal touch

The sense of touch, whether in the form of a hug, an arm clasp, or a pat on the shoulder, provides a powerful means of communicating, evoking, and modulating emotion. It does so through a complex communicative system that links toucher to touchee. Specifically, tactile communication takes place when the toucher intentionally alters the recipient’s perceptions, thoughts, feelings, or behavior through touch (Hertenstein, 2002).

How do we ascribe meaning to touch? The sense of touch arises from an array of mechanosensory structures within the layers of the skin. Mechanosensory neurons are specialized nerve cells that detect mechanical stimuli on the skin, such as variations in touch intensity, temporal dynamics, and the pressure of objects. These neurons are equipped with mechanoreceptors, which are sensory proteins or ion channels located in their membranes that respond to dimensions of tactile contact such as pressure, stretching, or deformation of surrounding tissue. As with other sensory modalities, mechanoreceptors generate electrochemical signals upon activation, which are transmitted from peripheral receptors to regions of the brain central to emotion, including subcortical areas such as the ventral striatum and amygdala, as well as the posterior insular cortex (PI) and the orbitofrontal cortex (OFC) (Björnsdotter and Olausson, 2011; Ellingsen et al., 2014; McGlone et al., 2012; Perini et al., 2015).

The forms of touch are myriad and often unfold as systematic sequences of actions, including, for example, stroking, rubbing, and squeezing (Hertenstein, 2002). Tactile acts like these can vary in intensity, velocity, abruptness, temperature, location, frequency, duration, and extent of the surface area touched. Across mammalian species, billions of skin cells and specialized neurons track dimensions of touch. These neurons send signals to regions of the brain, allowing the recipient to interpret and assign meaning to the tactile experience (Koelsch et al., 2018; Veldhuizen et al., 2022). For example, empirical studies find that perceptions of the pleasantness or unpleasantness of touch are related to physical characteristics of tactile contact, such as softness (Rolls et al., 2003), force and velocity (Löken et al., 2009), and temperature, namely warmth (Ackerley et al., 2014; Schepers and Ringkamp, 2010). Receptors within the layers of human skin can produce emotional responses (Valentini et al., 2012), either through the stimulation of erogenous areas, which evoke positive emotions, or through contact with nociceptive nerve endings, often resulting in pain and negative affect (Auvray et al., 2010).

Unmyelinated peripheral afferent fibers (C-touch, C-tactile, or CT fibers) project to the orbitofrontal cortex, anterior cingulate, anterior insula, and superior temporal sulcus, regions in which emotional stimuli are given social and affective meaning (Björnsdotter et al., 2014; Croy et al., 2016; Gordon et al., 2013; Li et al., 2019; Morrison, 2016; Olausson et al., 2002, 2010; Rolls et al., 2003). These specialized fibers located throughout the body function like a “social organ” (Morrison et al., 2010), responding to gentle and slow stroking within social interactions (Björnsdotter et al., 2010; Olausson et al., 2010; Wessberg et al., 2003). This stimulation initiates neurophysiological signals associated with reward and safety, such as elevated vagal tone and decreases in heart rate, blood pressure, and body temperature (Grandi and Gerbella, 2016; Grandi and Ishida, 2015). Moreover, the activation of CT fibers has been found to influence pleasantness ratings of touch, with variations depending on the firing frequency of afferents (Essick et al., 1999, 2010; Löken et al., 2009).

Another brain region activated by CT-targeted affective touch is the dorsal anterior cingulate cortex (dACC). In one relevant study, Gordon et al. (2013) reported that dACC showed greater connectivity with the left insula and amygdala during gentle touch on the arm. Activation of the dACC could play a role in ascribing meaning to touch-related stimuli given that this area is known to be involved in goal-directed action, which can also be construed as affect regulation (Gross, 2015; Gross and Thompson, 2007; Shenhav et al., 2017).

The most extensively studied neurochemical system that responds to social touch is the neuropeptide oxytocin, produced by neurons in the paraventricular nucleus (PVN) of the hypothalamus (Quintana et al., 2019). Tactile stimulation during social interactions and skin-to-skin contact can lead to the release of oxytocin, which is central to social engagement, emotion-related experiences, and information processing, such as attending to faces or perceiving emotional expressions (Bartz et al., 2011; Ellingsen et al., 2014; Leknes et al., 2013). Consistent with this, empirical studies on massage and parent-child skin-to-skin contact have demonstrated that the stimulation of CT afferents triggers oxytocin release (Grewen et al., 2005; Walker et al., 2017; Li et al., 2019; Morhenn et al., 2012; Matthiesen et al., 2001; Uvnäs-Moberg and Handlin, 2015; Uvnäs-Moberg and Petersson, 2010). Elevated oxytocin, in turn, is associated with neurophysiological shifts crucial for emotion regulation (Uvnäs-Moberg and Petersson, 2010), including increased heart rate variability (indicative of heightened vagal control; Kemp et al., 2012) and, more generally, increased parasympathetic autonomic nervous system activation (Gamer and Büchel, 2012). Building on this connection between touch, oxytocin release, and emotion regulation, a warm touch intervention with healthy married couples was found to increase salivary oxytocin while reducing stress-related markers, including blood pressure, plasma cortisol, and alpha-amylase, compared with a control group (Holt-Lunstad et al., 2008).

Taken together, recent advances in the study of touch reveal an interpersonal tactile system that involves specialized skin cells, oxytocin release, modulation of the peripheral nervous system, and the transmission of signals to subcortical (ventral striatum) and cortical regions of the brain, which are involved in the conceptual interpretation of emotion-related events (dACC and OFC; see Rolls et al., 2003). Our review highlights several ways in which touch contributes to emotion regulation: by downregulating negative emotions; by eliciting or upregulating positive states that counteract the physiological patterns of negative emotions (Fredrickson and Levenson, 1998; Ong and Allaire, 2005); by activating cortical regions involved in reappraisal processes, such as affect labeling (Ochsner and Gross, 2005); and likely by signaling safety, a condition that supports emotion regulation (Hefner et al., 2016).

## Touch and communication of emotional states

Emotion communication plays a crucial role in emotion regulation by signaling whether social contexts are safe or threatening. Such signals also indicate the appropriateness of specific affective responses and guide the selection of contextually fitting actions, both of which shape patterns of emotion regulation (Klinnert et al., 1986; Keltner and Kring, 1998; Marsh et al., 2007). Emerging research highlights the role of touch as a powerful medium for conveying distinct emotions (Hertenstein et al., 2006).

In an early demonstration of this thesis, Hertenstein and colleagues found that participants could decode anger, fear, disgust, love, gratitude, and sympathy at above-chance levels from brief touches by another participant to their arm, without seeing the toucher. For example, sympathy was associated with stroking and patting, anger with hitting and squeezing, disgust with a pushing motion, fear with trembling (Hertenstein et al., 2006), sadness with nuzzling and hugging, and love with hugging and stroking (Hertenstein et al., 2009). Touch can also influence the social interpretation of others’ facial expressions, enhancing the perceived friendliness and attractiveness of smiling faces while diminishing the perceived friendliness and attractiveness of angry faces (Ellingsen et al., 2014).

Developmental studies have documented that touch not only communicates distinct emotions in infants but also underpins the development of processes related to emotion regulation, such as affect labeling (Farroni et al., 2022; Walker and McGlone, 2013). Suggestive findings indicate that children can recognize an abrupt grasp as a signal of anger (Hertenstein, 2002) and interpret a mother’s firm hold as an expression of distress, which also shapes their responses to novelty (Hertenstein and Campos, 2001). For example, one recent study showed that 7-month-old infants who received affective touch (versus non-affective stimulation) showed less avoidance of an angry expression, whereas those who received non-affective stimulation still looked away. This suggests that gentle stroking can help babies be more confident and explore their surroundings more (Addabbo et al., 2021), which may support approach-oriented regulatory processes. Although these studies primarily examine how touch influences infants’ initial appraisal of stimuli, they also highlight its role in reappraisal processes and the regulation of emotional responses, including suppression or acceptance of emotions (Mauss and Gross, 2004; Mauss and Tamir, 2013; Shallcross et al., 2010; Shallcross et al., 2015). For example, receiving touch-related expressions of anger or sympathy can communicate alternative interpretations of an external emotional stimulus, which is central to emotion regulation (Gross et al., 2011; Tamir, 2016). Taken together, we theorize that the communication of emotion through touch can guide emotion regulation, a thesis we expand on in this review.

## Functions of touch

Touch plays a profound role in structuring social interactions (Cascio et al., 2019). For example, it can impact individuals’ inclination to comply with requests, express mutual trust and support, alter attitudes towards others, foster the creation of bonds within couples and groups, and help manage stress (Dunbar, 2010, 2018; Hostinar et al., 2014; Shutt et al., 2007). Below, we identify four functions of touch, all of which likely play a role in regulatory processes.

### Eliciting cooperation

Cooperation—the collaboration for mutual gain (and avoiding the costs of conflict)—is vital to social interactions (Rand and Nowak, 2013). Numerous studies have documented the role of touch in fostering cooperation and, more broadly, prosocial behavior. Illustrative studies have shown that participants are more likely to comply with a request when accompanied by touch (Willis and Hamm, 1980), return a dime left in a phone booth if the previous person touched them rather than if they had not been touched (Kleinke, 1977), and volunteer to participate in class exercises when a teacher touches them on the forearm (Guéguen, 2004).

In a line of research conducted in restaurant settings, studies documenting what is known as the “Midas touch” effect examined how brief and incidental touch by waitresses on either the hand or the shoulder modulates customer behavior relative to a no-contact control condition. Customers who experienced tactile contact showed a higher propensity to tip (Crusco and Wetzel, 1984; Erceau and Guéguen, 2007; Stephen and Zweigenhaft, 1986). In a similar study, bus riders were more willing to give a passenger a free ride if that passenger touched them while making the request (Guéguen and Fischer-Lokou, 2003). Likewise, research on spontaneous touch among NBA teammates highlights its critical role in team functioning. Systematic coding of touch gestures—such as high fives and fist bumps—revealed that greater touch frequency, averaging approximately two seconds per player per game, was linked to increased cooperation (e.g., defensive support, effective passing) and improved overall team performance by the season’s end (Kraus et al., 2010).

These findings suggest that touch serves as a strong medium for communicating cooperation, prosocial intention, and concern (Lazar and Eisenberger, 2022; Penner et al., 2005). In this way, touch helps create contexts of perceived social support, which are central to stress regulation (Berkman and Kawachi, 2000; Bolger et al., 2000). Through this function, individuals may facilitate more effective downregulation of emotions in others, a hypothesis that warrants further empirical investigation.

### Signaling safety

Touch can also function as a powerful cue for signaling safety, shaping emotional and social development across the lifespan. This insight first emerged in the attachment literature, where researchers centered upon processes by which infants gather information from parents about the safety (or peril) of the social context (Hertenstein and Campos, 2001; Main, 2013).

Attachment theory suggests that children who receive touch that conveys the message of safety and security are predisposed to developing secure attachement later in life (Anisfeld et al., 1990; Weiss et al., 2000), which is known to be associated with a greater capacity to reappraise (Troyer and Greitemeyer, 2018; Domic-Siede et al., 2024; Karreman and Vingerhoets, 2012). For example, infants who were held tenderly and for longer periods of time, compared with those who were held reluctantly or awkwardly, were more securely attached (Ainsworth et al., 2015). Experimental and correlational evidence indicates that infants who experience more frequent touch, such as being held in soft baby carriers rather than car seats, are more likely to develop secure attachment styles, a gateway to specific emotion regulation strategies, such as reappraisal (Weiss et al., 2000). These benefits of touch extend beyond infancy into childhood and adolescence. For example, parental touch for children (ages 8–10) and adolescents (ages 11–14) has been shown to reduce social vigilance and foster trust, particularly among socially anxious children. Furthermore, parental touch correspondingly reduced children’s attention to angry faces, emphasizing its protective function against threatening stimuli (Brummelman et al., 2019). These studies clearly show how parental touch—affiliative and loving kinds, of course—enables an attachment style that promotes reappraisals of threatening stimuli.

In adults, touch also plays a vital role in signaling safety and mutual trust (Dunbar, 2010, 2018; Hostinar et al., 2014), which supports the formation and maintenance of close relationships (Baumeister and Leary, 1995). It communicates responsiveness, acceptance, and warmth (Collins and Feeney, 2004; Jones and Yarbrough, 1985), fostering a sense of security that reduces defensive behaviors (Mikulincer et al., 2013). For example, during shared activities, such as watching television, touch heightens perceptions of security, strengthening mutual understanding, care, and interpersonal closeness (Jakubiak and Feeney, 2016a; Debrot et al., 2013). Furthermore, even imagining touch experiences can enhance access to security-related concepts, such as calmness, support, and trust (Jakubiak and Feeney, 2016b).

Empirical evidence suggests that safety cues facilitate the downregulation of fear and anxiety (Hefner et al., 2016). Furthermore, akin to the effects of cooperation, signals of safety could also enhance successful emotion regulation by fostering perceived responsiveness and availability. Therefore, we propose that touch can be harnessed as an emotion regulation tool by directly activating safety signals in the recipient, thereby activating the aforementioned pathways (e.g., trust, responsiveness, acceptance, warmth, etc.), which in turn may increase the likelihood of successful emotion regulation, most notably through reappraisal.

### Rewarding behavior

A third function of touch is to communicate reward, as reflected in the pleasurable experience that arises from the stimulation of CT afferents (Gordon et al., 2013; Lindgren et al., 2012; Nummenmaa et al., 2016; McGlone et al., 2012; Walker and McGlone, 2013). In the social domain, the rewarding aspects of touch can manifest in various forms. These can range from gentle stroking among close friends and parent–child interactions to celebratory high-fives between teammates (e.g., Kraus et al., 2010). In addition, a pat on the shoulder can be rewarding as it conveys the meaning of reinforcement, and a caress between romantic partners may provide pleasure.

We posit that touch, through the activation of the orbitofrontal cortex (a reward-related region) and the release of oxytocin (Lamm et al., 2015), may enable emotion regulation. Furthermore, the activation of pleasure, which we can also call activation of positive affect, can downregulate negative emotions, as has been widely studied in the emotion regulation literature (e.g., Fredrickson and Levenson, 1998).

### Soothing pain and stress

Perhaps most relevant to emotion regulation, touch can alleviate both physical and psychological distress (Ellingsen et al., 2016). This calming effect can be seen as a crucial element in the process of managing and modulating emotional responses.

Touch can contribute to alleviating physical pain, notably through the activation of C-tactile fibers (Gursul et al., 2018; Liljencrantz et al., 2013). For example, in a study examining infant responses to a heel lance procedure, infants receiving maternal skin-to-skin contact exhibited an 82% reduction in crying and a 65% reduction in facial grimacing compared with a control group, alongside a lower heart rate throughout the procedure. Adult studies further illustrated the impact of touch on modulating pain responses. For example, an fMRI study examined the neural responses of 16 married women exposed to the threat of electric shock while holding either their husband’s hand, the hand of a male experimenter, or no hand at all (Coan et al., 2006). Holding their husband’s hand led to a pervasive reduction in activation in neural systems associated with threat responses, as did holding a stranger’s hand (although the effects were not as robust).

Studies of brain-to-brain coupling during experiences of pain showed that handholding was associated with self-reported analgesia (Goldstein et al., 2018). Additionally, handholding during negative emotional experiences decreased activation in affective-pain-related neural regions, such as the anterior cingulate cortex and anterior insula (Kraus et al., 2019). This effect extends beyond physical pain to emotional pain. Specifically, handholding has been found to lessen the emotional pain of recalling negative emotional experiences (Sahi et al., 2021).

This stress-alleviating effect of touch can be observed across a variety of species. Starting with non-human primates, grooming reduces heart rate and displacement activities related to stress, such as striking others (Aureli et al., 1999). Similarly, maternal licking and grooming in rats during the first postnatal week are crucial for attachment formation and stress regulation (Bagot et al., 2012; Kaffman and Meaney, 2007). Male rats reared by dams with high contact exhibit diminished stress responses, heightened exploratory tendencies in novel environments, and enhanced cognitive performance (Caldji et al., 2000). This is also evidenced by findings that rat pups receiving high levels of maternal contact exhibit a range of benefits, including reduced activity in stress responses and lower corticosterone (a stress-related hormone), both immediately and when they are mature (Francis et al., 1999; Levine and Stanton, 1984; Meaney, 2001).

The stress-buffering effect of touch is also evident in humans, beginning in infancy. For example, infants receiving affectionate touch during a stressful task are more likely to be protected from the psychological and physiological effects of stress (Feldman et al., 2010; Jean et al., 2009). In addition, experimental evidence indicates that premature infants assigned to get skin-to-skin contact with their caregivers (versus those who received standard care) exhibit attenuated stress responses as measured by cortisol levels, even 10 years later (Feldman et al., 2014).

In adults, correlational evidence indicates that greater affectionate touch is linked to lower self-reported stress and reduced salivary cortisol levels (e.g., Burleson et al., 2007), whereas touch avoidance may increase vulnerability to stress-related disorders (e.g., Burleson et al., 2007; Stevens et al., 2024). Building upon that, experimental studies showed that women who experienced touch with their partner before a standardized psychological laboratory stressor exhibited significantly lower cortisol and heart rate responses compared with those who received social support or no social interaction (Ditzen et al., 2007). Similarly, individuals who reported receiving more frequent hugs from their partners in their normal day-to-day interactions had smaller heart rate increases during a stressful laboratory task than individuals who reported less frequent hugs (Light et al., 2005). Furthermore, it was shown that engaging in affectionate touch while discussing a positive relational experience before a stressful speech task reduced heart rate and blood pressure reactivity (Grewen et al., 2003).

In the context of stress, touch not only buffers against upcoming stressors but also facilitates recovery from them. For example, greater amounts of touch received after a stressful laboratory task predicted greater reductions in distress (Robinson et al., 2015). Even recalling and imagining supportive touch proved effective in diminishing perceived stress of a socially evaluative task and attenuated stress more effectively than imagining instances of a partner verbally expressing support (Jakubiak and Feeney, 2017).

In summary, emotion regulation fundamentally involves the downregulation of negative states, including pain, anger, fear, and distress. The studies we have reviewed here robustly reveal that the right kind of touch can reduce stresses of social and physical kinds across species, clearly showing how touch is involved in emotion regulation.

## Touch as emotion regulation

Emotion regulation involves the activation of goals oriented toward altering an unfolding emotion-related trajectory (Gross et al., 2011; Tamir, 2016), which includes attempts to influence the duration, intensity, and quality of emotions (Naragon-Gainey et al., 2017). Decades of scholarship have characterized the intrapersonal benefits of emotion regulation (Gross et al., 2006; Gross and John, 2003). More recently, scientists have noted that emotion regulation is often interpersonal, involving interactions in which people modulate both their own and others’ emotional responses (Butler and Gross, 2009; Coan et al., 2006; Rimé, 2007). Building upon our review, we argue that touch is central to interpersonal emotion regulation, as it also alters one’s own and others’ emotion trajectories by shifting emotion-related physiology, appraisals, experience, and conceptualization. With the aim of stimulating future research, we now offer a framework for illuminating the processes by which touch supports emotion regulation.

### Touch supports emotion regulation by activating emotion-generative regions of the recipient’s brain

The first pathway by which interpersonal touch can support emotion regulation is through its direct effects on the recipient’s central and peripheral nervous system. For example, one line of inquiry suggests that touch activates the insular cortex, which may help modulate autonomic responses (Jönsson et al., 2018; Tuulari et al., 2019). Touch likewise can activate regions of the prefrontal cortex (specifically, the orbitofrontal cortex), which is also involved in the downregulation of negative emotions (Etkin et al., 2015; Ochsner and Feldman Barrett, 2001). Handholding reportedly increases functional connectivity between pain-related regions and medial prefrontal cortices, which is also suggestive of regulatory processes (López-Solà et al., 2019). This evidence suggests that receiving touch might help people reappraise the intensity of negative emotions and pain by increasing prefrontal activity (Shamay-Tsoory and Eisenberger, 2021). Along similar lines, interpersonal touch leading to certain neurophysiological changes we considered earlier, such as the release of oxytocin, may help downregulate cortisol release after stressful events (Ditzen et al., 2009). These findings suggest how touch can facilitate emotion regulation in interpersonal contexts through its direct effects on neurophysiological processes.

### Touch supports emotion regulation by enhancing emotional awareness and facilitating the labeling of unfolding emotions and their contexts

Central to emotion regulation are interpretive processes through which individuals label the emotional meanings of unfolding social events (Lieberman et al., 2007). Touch, we posit, can enable individuals to label, or conceptualize, their ongoing emotional experiences. More specifically, as with facial and vocal expressions, touch provides recipients with emotion-related concepts to label the present context.

Empirical evidence shows that even a simple touch, such as contact on the arm, can activate an array of emotion concepts in the recipient. For example, parents can rely on touch to convey emotion-related interpretations to infants about individuals and events in the environment, signaling whether they are frightening or affiliative (Hertenstein and Campos, 2001; Hertenstein et al., 2006). Through this process, touch facilitates the recipient’s ability to label or conceptualize an emotional experience within its contextual framework. This allows them to appraise the unfolding emotional events and thereby regulate their emotions.

The importance of labeling emotions becomes even more relevant in the context of emotion regulation. Ample empirical evidence supports the proposition that labeling one’s own or others’ emotions can facilitate a reduction in negative emotional experiences and, therefore, can be conceptualized as an implicit form of emotion regulation (Wilson and Schooler, 1991; Torre and Lieberman, 2018). Specifically, affect labeling has demonstrated less amygdala activity than perceptual processing of the emotional aspects of the same image (Lieberman et al., 2007), indicating its potential to downregulate evocative components of the stimuli.

Our theorizing converges with the idea that recognition of an emotion or emotion-eliciting event can facilitate many forms of emotion regulation (Izard et al., 2001; Lane and Nadel, 2000). We contend that touch may be a primary means through which individuals recognize emotions and related events in social interactions, a function that is particularly prominent in early development. Given this, future studies should investigate whether touch facilitates the recognition of the recipients’ emotional experiences (e.g., distress or threat), thereby aiding more effective regulation.

### Touch supports emotion regulation by communicating relational appraisals of social contexts

Emotions arise out of primary evaluations of the social context, which shape how individuals interpret and respond to their environment (Frijda, 1986; Keltner et al., 2022; Lazarus, 1991). We propose that touch aids emotion regulation by communicating relational appraisals of social interactions, such as signals of cooperation, safety, and reward, as well as shaping the emotions that ensue within the relational context.

First, cooperation is a primary relational appraisal of social interaction and is shaped by touch, as noted earlier. We propose that cooperation facilitates emotion regulation by offering emotional and instrumental support and by eliciting affiliative emotions (Collins and Read, 1990; Uchino, 2009; Van Kleef et al., 2004a; Van Kleef et al., 2010). It is also interesting to speculate how touch-initiated appraisals of cooperation foster a sense of inclusion within groups, enabling individuals to regulate threat-related emotions more effectively (Coan and Sbarra, 2015). Therefore, we reason that specific kinds of touch generate appraisals of cooperation and support within social interactions, aiding emotion regulation (Gable et al., 2004; Gross, 1998; Rimé, 2009).

Second, empirical evidence shows that touch reduces negative affect by fostering feelings of safety and security—another relational appraisal of social interactions (Anisfeld et al., 1990; Hertenstein and Campos, 2001; Jakubiak and Feeney, 2017; Mikulincer and Shaver, 2007). Either directly or as a conduit for these feelings, touch also communicates acceptance, diminished feelings of exclusion, trust, availability, responsiveness, care, and intimacy, all of which enhance perceived support (Baldwin, 1992; Carnelley and Rowe, 2007; Coan et al., 2017; Collins and Feeney, 2004; Debrot et al., 2012; Gillath et al., 2008; von Mohr et al., 2018).

Our proposition that touch supports emotion regulation by stimulating security—and, in turn, conveying acceptance, availability, responsiveness, and other indicators of social support—has found expression in the stress-buffering hypothesis, a conceptual framework suggesting that perceived support may facilitate stress reduction (Cohen and Wills, 1985). For example, the amount of touch a child receives is often used as an index of maternal availability, a crucial factor in shaping emotion regulation early in life (Feldman et al., 2011). Similarly, in adults, individuals who expect their partners to be available and supportive tend to be less reactive to stress, as this perceived support inevitably shields them against stress (Uchino et al., 1996; Ditzen et al., 2007; Kane et al., 2012). In addition, people primed with reminders of closeness and security can regulate their affect more effectively than individuals who do not experience these primes (Gillath et al., 2008; Selcuk et al., 2012). Synthesizing these lines of research, it is evident that touch gives rise to relational appraisals and fulfills an affiliative function that reinforces social bonds, ultimately contributing to stress reduction, a central objective of emotion regulation (Sandstrom and Dunn, 2014; Jakubiak and Feeney, 2017).

Third, the reward system may be another plausible pathway through which touch can lead to reduced stress. Touch can be rewarding in many ways, including affirmation, approval, praise, and care, all of which can alleviate stress (Creswell et al., 2005). Similarly, studies with humans and non-human species have found that activation of the brain’s reward system might be directly linked to reductions in stress measured by multiple stress outcomes such as physiological stress responding, distress to pain, and stress behaviors (Dutcher, 2023; Ulrich-Lai and Herman, 2009; Ulrich-Lai et al., 2007; Ulrich-Lai et al., 2010). The rewarding properties of touch likely play a role in several forms of emotion regulation, including reducing the intensity of negative affect and cortisol levels (Eisenberger et al., 2011; Inagaki and Eisenberger, 2012; Morelli et al., 2014; Seehausen et al., 2012) as well as serving as a distraction from distressing stimuli (Shamay-Tsoory and Eisenberger, 2021).

Taken together, we suggest that understanding the influence of touch on primary appraisals of cooperation, safety, and reward, and the ways in which such appraisals shape emotion regulation, is a fertile area for future inquiry. In keeping with this possibility, Debrot et al. (2013) provided initial evidence by testing psychological intimacy as a potential mediator through which touch enhances effective emotion regulation, as demonstrated in a daily diary study within the context of romantic relationships. Future research should build upon this work by focusing on our proposed pathways and mechanisms linking touch to stress reduction, thus revealing touch as a form of emotion regulation.

### Touch supports emotion regulation by regulating distress and negative affective states

A central focus of emotion regulation is the reduction of distress, such as pain stemming from trauma, stress, or conditions like anxiety and depression. A recent meta-analysis by Packheiser et al. (2024) examined the efficacy of touch interventions across a range of mental and physical health conditions. Their findings revealed a medium effect size for touch in reducing pain, depression, and anxiety in both children and adults, underscoring the broad applicability of touch-based interventions for downregulating distress.

Beyond its capacity to co-regulate emotions with others, touch can also be a powerful tool for self-regulation of distress. Recent research indicates that a brief 20-second compassionate self-touch can effectively reduce one’s own stress (Susman et al., 2024). These findings highlight the potential of touch interventions as an easily accessible emotion regulation tool for managing both one’s own and others’ emotional states.

While the link between touch and stress reduction is widely accepted, researchers have only recently begun directly linking touch and emotion regulation. For example, Burleson et al. (2022) introduced the concept of “touch for affect regulation (TAR).” They observed the effects of this technique in individuals who consciously used touch to regulate their emotions and found that those who employed touch as an emotion regulation tool were more likely to benefit from affectionate touch.

In addition to its soothing effects on pain and negative emotions (Coan et al., 2006; Liljencrantz et al., 2013; Mancini et al., 2014, 2015) and its direct impact on stress reduction, touch may also facilitate emotion regulation through specific strategies, such as reappraisal or distancing. For instance, the soothing aspect of touch might allow individuals to reappraise the intensity of their emotions and the situation itself, enabling them to perceive the situation as less distressing. A parent may tickle a child to encourage them to reappraise a sullen mood. Similarly, in the midst of an argument or conflict, a romantic partner may apply a soothing touch to encourage awareness of reappraisals of current emotions. Alternatively, this touch may help direct attention to the soothing stimuli, effectively distracting from sources of distress. Future research should investigate how these distinct pathways connect touch to self- and other-regulation of emotion.

## Future directions

Throughout our review, we have explored the multifaceted ways in which touch can function as a form of emotion regulation, tool highlighting its role in modulating neurophysiological processes, influencing contextual interpretations and relational appraisals, alleviating both physiological and psychological pain, and, crucially, attenuating stress responses. Building on this framework, we now turn our attention to the potential connections between touch and classic emotion regulation theory and research.

The emotion regulation process involves intrinsic regulation, where the goal is to regulate one’s own emotions, as well as extrinsic emotion regulation, where the goal is to regulate another person’s emotions (Gross, 2015). As individuals engage in these goal-directed processes, people adeptly employ a diverse range of strategies in everyday life. However, the literature on emotion regulation has historically been dominated by work on two strategies: reappraisal and suppression. Recent theoretical perspectives suggest that engaging in these regulatory efforts might be costly because they require mental effort, and that sensory modalities, including touch, offer a more effortless route to emotion regulation by inducing immediate changes in neurophysiology (Rodriguez and Kross, 2023).

In this review, we propose that touch can reduce the cost of engaging in emotion regulation strategies. For example, in situations requiring extrinsic emotion regulation, if a mother soothes her distressed child through touch, or if a friend offers a hug or touches an arm to comfort someone in distress, the inherent soothing effect of touch may render reappraisal unnecessary, as the distress is already alleviated. Recent research speaks to this possibility, suggesting that touch can be more powerful than verbal support in interpersonal emotion regulation. Specifically, studies have shown that touch can reduce physiological reactivity to stressors better than verbal support (Ditzen et al., 2007), highlighting its potential as a more effortless route for downregulating distress. This empirical evidence can guide research examining the interaction between classic emotion regulation strategies and touch in order to develop this line of inquiry further.

Research on emotion regulation strategies has revealed that their adaptiveness varies depending on contextual factors, such as the intensity and the controllability of a situation (Bonanno and Burton, 2013; English et al., 2017). Specifically, reappraisal tends to be more effective in uncontrollable situations compared with controllable ones (Troy et al., 2013), while distancing, relative to reappraisal, is more effective and preferred in high-intensity situations (Sheppes et al., 2011; Sheppes et al., 2014; Sheppes and Meiran, 2007). Examining the effectiveness of touch as an emotion regulation tool across diverse contextual factors could offer valuable insights into whether it indeed provides more effortless ways to alter the occurrence, intensity, and expression of emotional states appraised as unfitting to the present context.

## Conclusion

Across the lifespan, from infancy to old age, touch remains a powerful force in shaping our emotional landscape. As the field of emotion regulation increasingly emphasizes interpersonal dynamics, we argue that the role of touch emerges as a primary and often under-appreciated form of regulation. To make this case, this review has highlighted the neurophysiological underpinnings of touch, revealing its profound impact on emotion processing. We have outlined four core functions of touch—eliciting cooperation, signaling safety, activating reward systems, and reducing distress—each with implications for how touch shapes emotional experience. From its direct effects on neural circuitry to the symbolic meanings embedded in touch, the evidence underscores the robust regulatory capacity of this often overlooked modality in social interaction. These pathways, through which touch may function as a form of emotion regulation, may open fruitful avenues for empirical investigation.
